# Salmonella osteomyelitis in a previously healthy neonate: a case report and review of the literature

**DOI:** 10.1186/s13052-018-0464-2

**Published:** 2018-02-26

**Authors:** Canyang Zhan, Jing Du, Lihua Chen

**Affiliations:** Department of neonatology, Children’s Hospital, School of Medicine, Zhejiang University, Binsheng Road 3333, Hangzhou, 310003 People’s Republic of China

**Keywords:** *Salmonella*, Osteomyelitis, Neonate

## Abstract

**Background:**

Acute osteomyelitis, which is uncommon in neonates, needs to be quickly diagnosed and treated to avoid devastating sequelae. Therefore, it is important to maintain a high index of suspicion and be aware of the evolving epidemiology and of the emergence of antibiotic resistant and aggressive strains requiring careful monitoring and targeted therapy. The most frequently isolated bacterium in neonates with osteomyelitis is *Staphylococcus aureus*, while *Salmonella* is an unusual organism causing osteomyelitis and is exceedingly rare in non-sickle cell disease children.

**Case presentation:**

We report an extremely rare case of neonatal osteomyelitis caused by *Salmonella* in a neonate, who was previously healthy. We report this case because it was caused by a rare pathogenic germ in newborns and by its non-specific presentation.

**Conclusions:**

*Salmonella* should be kept in mind in the differential diagnosis of neonatal osteomyelitis. It is important to start antibiotic therapy as soon as possible and to adjust therapy in relation to the susceptibility of the bacterial strain.

## Background

Acute osteomyelitis is uncommon in neonates. Any bacteria can cause osteomyelitis in children. The most frequently isolated bacterium in neonates with osteomyelitis is *Staphylococcus aureus*, accounting for more than 50% of cases. Other microorganisms include *Escherichia coli, Streptococcus*, *Klebsiella* and *Proteus* [1, 2]. In general, *Salmonella* rarely causes osteomyelitis in the newborn or in non-sickle cell disease children. In this paper, we presented a case of *Salmonella* osteomyelitis in a previously healthy neonate.

## Case presentation

**Fig. 1:**
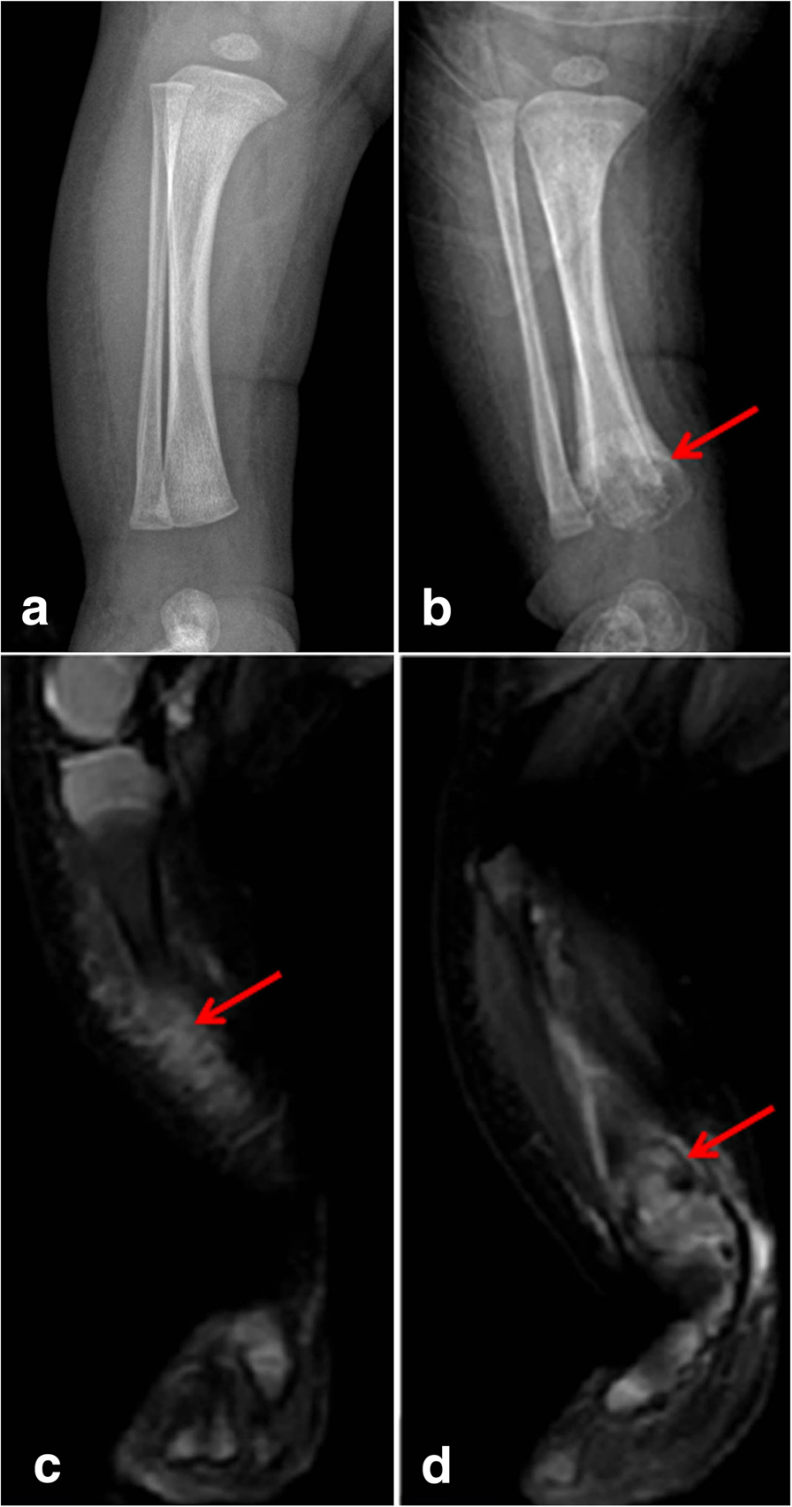
The radiography of the right lower limb on admission (**a**) compared with the radiography of the same limb at diagnosis (**b**) shows the destruction of the distal tibia, and the periosteal reaction (red arrow). The MRI of the right lower limb shows significant edema of the soft tissue (**c**), the destruction of the distal tibia and the periosteal reaction of the tibia and fibula (**d**) (red arrow)

A 28-day-old neonate presented with excessive crying and fever, and without diarrhea, stipsis or vomiting. Then he was admitted into the neonatal department. The baby was previously healthy and was fed a mixed diet (breast milk and milk powder). His mother usually used 40 degrees Celsius tap water, which had been boiled, to brew the milk powder. Additionally, he had no family history of sickle-cell disease. Physical examination found a slight swelling of the right lower limb. Blood tests showed the following: white blood cell count (WBC): 11430 cells/μl, neutrophils: 55.7%, hemoglobin: 133 g/l, C-reactive protein (CRP): 2.1 mg/dl, and HIV testing: negative. The serum concentration of procalcitonin was 0.405 nanograms/ml. The cytokine examination, including IL-2, IL-4, IL-6, IL-10, TNF, and IFN-γ, showed a high level of IL-6 (109.3 picograms/ml), IL-10 (8.9 picograms/ml), and IFN-γ (27.4 picograms/ml). An ultrasound scan of the right lower limb showed mild edema of the surrounding soft tissue. Radiographs of the right limb at admission were normal (Fig. 1a). Thus, the baby was treated with antibiotics (ampicillin sulbactam) for suspected sepsis. After 4 days, his fever persisted without improvement of the inflammatory markers. From the blood culture, which was taken before administration of antibiotic therapy, *Salmonella typhi* developed and the strain was resistant to ampicillin sulbactam (MIC ≧ 32 micrograms/ml). According to the pathogen susceptibility results, the antibiotic was changed, and the meropemen, 90 mg every 8 h intravenously, was started (MIC ≦ 0.5 micrograms/ml). Unfortunately, the microbiological cultures were not carried out on milk. The baby showed rapid clinical improvement, and normalization of inflammatory markers. However, the swelling of the right lower limb worsened. After an orthopedic consultation, radiographs of the right lower limb were ordered once again, and showed destruction of the distal tibia on the 10th day after admission (Fig. 1b), confirming the clinical suspicion of osteomyelitis. MRI of the right lower limb on the 26th day after admission showed significant edema of the soft tissue (Fig. 1c) and destruction of the distal tibia and periosteal reaction of the distal tibia and fibula (Fig. 1d). After treatment with meropenem for approximately 3 weeks, the baby was discharged in good condition. During the follow-up exam 3 months after discharge, no recurrence and sequelae were observed. Meanwhile, there were no manifestations of anemia and immune deficiency during follow-up.

## Discussion

There are different clinical symptoms of *Salmonella* infections, including the following: gastroenteritis, enteric fever, bacteremia and focal disease (including soft tissue infection). Moreover, *Salmonella* can persist in the bowel after an infection, when the clinical symptomatology of the acute infection has disappeared and generates the condition of chronic colonization, or a chronic carrier. A resulting *Salmonella* infection was one of the most common food-borne infections, caused from contaminated water, milk and food. It was also reported that the transmission was associated with reptiles. For infants, ingestion of contaminated formula is a significant risk factor associated with *Salmonella* infection [2]. Case control studies suggested that breast-feeding was a strong protective factor against acquiring *Salmonella* infection during infancy [3]. In this study, although the baby was fed a mixed diet (breast milk and milk powder), the route of infection was unclear. Through further questioning of the history of feeding, it was determined that the formula might have been stored inappropriately during summer and the baby might have become infected from contaminated formula.

*Salmonella* osteomyelitis is very rare in normal children, amounting to 0.45% of all osteomyelitis cases. The infection is usually associated with hemoglobinopathies, such as sickle-cell disease, malignancies and immune deficiencies [4]. There are few reports about *Salmonella* osteomyelitis in healthy individuals in English. From 1978 to 2012, 21 cases of *Salmonella* osteomyelitis in healthy children have been reported in the United States. In the present case, the patient was younger than 1 month. He was previously healthy and showed no manifestations of anemia and immunosuppression. To our knowledge, the report of *Salmonella* osteomyelitis in a healthy neonate is very rare. Regarding these cases in China, there are no detailed national data about the incidence of neonatal *Salmonella* and neonatal osteomyelitis. However, sporadic case reports show that the incidence of *Salmonella* infections in neonates ranges from 1 to 2 cases for every 1000 pediatric hospital admissions, while neonatal osteomyelitis accounts for approximately 2–4% of all pediatric osteomyelitis cases in China.

*Salmonella* osteomyelitis is clinically and radiologically indistinguishable from osteomyelitis caused by other organisms. Moreover, the signs of osteomyelitis in plain radiographs usually do not appear until 10 to 14 days after onset of symptoms [5–7]. Diagnosis is often delayed during the acute phase. With regards to our case, the radiographs were normal on admission and the osteomyelitis feature was found 2 weeks after the onset of disease. More attention should be paid to neonates with local swelling or redness and osteomyelitis should be considered. Moreover, it is suggested that MRI is helpful for diagnosing osteomyelitis earlier compared to radiographs [8]. In early osteomyelitis, bone marrow edema with a low signal on T1- and a high signal on T2-weighted images are visible. Gadolinium injection results in an important signal enhancement of the infected marrow. Therefore, we suggest that it is better to perform MRI during the start of symptoms if the radiographs and ultrasound are silent.

Adjusting antibiotic therapy, according to the results of the culture, is very important for *Salmonella* osteomyelitis. Usually, *Salmonella* is sensitive to fluoroquinolones, third generation cephalosporins and penicillin antibiotics [9]. The case reported by Saturveithan C was sensitive to ampicillin [8]. In our study, the susceptibility results for *Salmonella* showed that it was sensitive to ceftriaxone and meropenem, while it resistant to ampicillin sulbactam. The total duration of antibiotic administration was 28 days (ampicillin sulbactam 4 days and meropenem 24 days). There was no optimal suggestion about the duration and routes of antibiotic administration for *Salmonella* osteomyelitis in neonates.

## Conclusions

In conclusion, *Salmonella* should be kept in mind in the differential diagnosis of neonatal osteomyelitis. It is important to start antibiotic therapy as soon as possible and to adjust therapy in relation to the susceptibility of the bacterial strain.

## Abbreviations

CRP: C-reactive protein
WBC: white blood cell
